# A rapid method for post-antibiotic bacterial susceptibility testing

**DOI:** 10.1371/journal.pone.0210534

**Published:** 2019-01-10

**Authors:** Andrew A. Heller, Dana M. Spence

**Affiliations:** 1 Department of Chemistry, Michigan State University, East Lansing, Michigan, United States of America; 2 Institute for Quantitative Health Sciences & Engineering, Michigan State University, East Lansing, Michigan, United States of America; 3 Department of Biomedical Engineering, Michigan State University, East Lansing, Michigan, United States of America; Universidade de Aveiro, PORTUGAL

## Abstract

Antibiotic susceptibility testing is often performed to determine the most effective antibiotic treatment for a bacterial infection, or perhaps to determine if a particular strain of bacteria is becoming drug resistant. Such tests, and others used to determine efficacy of candidate antibiotics during the drug discovery process, have resulted in a demand for more rapid susceptibility testing methods. Here, we have developed a susceptibility test that utilizes chemiluminescent determination of ATP release from bacteria and the overall optical density (OD600) of the bacterial solution. Bacteria release ATP during a growth phase or when they are lysed in the presence of an effective antibiotic. Because optical density increases during growth phase, but does not change during bacterial lysing, an increase in the ATP:optical density ratio after the bacteria have reached the log phase of growth (which is steady) would indicate antibiotic efficacy. Specifically, after allowing a kanamycin-resistant strain of *Escherichia coli (E*.*coli)* to pass through the growth phase and reach steady state, the addition of levofloxacin, an antibiotic to which *E*. *coli* is susceptible, resulted in a significant increase in the ATP:OD600 ratio in comparison to the use of kanamycin alone (1.80 +/- 0.50 vs. 1.12 +/- 0.28). This difference could be measured 20 minutes after the addition of the antibiotic, to which the bacteria are susceptible, to the bacterial sample. Furthermore, this method also proved useful with gram positive bacteria, as the addition of kanamycin to a chloramphenicol-resistant strain of *Bacillus subtilis (B*. *subtilis)* resulted in an ATP:OD600 ratio of 2.14 +/- 0.26 in comparison to 0.62 +/- 0.05 for bacteria not subjected to the antibiotic to which the bacteria are susceptible. Collectively, these results suggest that measurement of the ATP:OD600 ratio may provide a susceptibility test for antibiotic efficacy that is more rapid and quantitative than currently accepted techniques.

## Introduction

The cost of a molecule drug candidate to reach market is currently estimated at $2.5 billion, while the time to bring that drug to market is approximately 10 years.[1] The cost is often a result of only 10% of experimental drugs that enter clinical trials reach approval by the Food and Drug Administration (FDA).[2] The amount of time required for a drug to reach approval may also be due to an increase in the frequency of procedures performed during clinical protocols.[3] In either case, potential drugs that fail during late-stage clinical trials cost more than those failing at earlier stages of the drug development process.[4] The increasing cost and time of drug development have led to concerns related to declining economic returns for new drugs,[5] which may be a determinant in the decrease in number of approved drugs despite continued resources being dedicated to new drug discovery.[2] Thus, while a definitive reason for the decreasing number of new drugs is a topic of debate,[6–8] it is evident there are less new drugs being approved and, in turn, less new drugs reaching the public.[9] This trend in approved drugs certainly applies to antibiotics for fighting bacterial infections.

Bacteria have the ability to quickly evolve while under selective pressure caused by a given antimicrobial agent, therefore they have a better chance of survival from future exposure to that antimicrobial agent, a phenomenon known as antimicrobial resistance. A subclass of antimicrobial resistance caused by antibiotics is known as antibiotic resistance, which has been increasing in frequency and number since the discovery of penicillin in 1940.[9–11] A danger of antibiotic resistance is that bacteria have the ability to transfer antibiotic resistant genes to other bacteria via a process known as horizontal gene transfer,[12–15] which has led to the formation of pandrug resistant bacteria strains that are resistant to all current antibiotics.[16–18] The imminent threat of antibiotic resistant bacteria led to the release of an executive order for combating antibiotic resistant bacteria.[19, 20] The result of the executive order was the formation of the National Action Plan, which consisted of five goals to combat antibiotic resistant bacteria, including rapid diagnostic tools and tools for accelerating research in the development of antibiotics and therapeutics.[21] Both of these goals rely heavily on susceptibility tests.

A susceptibility test involves challenging bacteria with an antibiotic and determining if the bacteria can grow in the presence of the antibiotic. The three most common methods are the broth microdilution method, the disk diffusion method, and the Etest method.[22] The broth microdilution method is the most common method and involves diluting antibiotics in growth media to create a series of antibiotic solutions, each with a smaller concentration of antibiotic. Bacteria are inoculated into the antibiotic-media solution and incubated overnight to promote growth. The type and concentration of antibiotic could be determined from this test for a given bacteria strain.[22] The broth microdilution assay can be adapted for the high throughput of samples or for colorimetric readout for other microbial species; however, a period of 24–48 hours is required to allow for bacteria growth.[23–25] The disk diffusion method utilizes the diffusion of antibiotics through an agar plate to create a gradient of decreasing antibiotic concentration as distance from the antibiotic source increases. Bacteria are grown on the plate and the distance of growth from the source of the antibiotic can be used to determine if the bacteria are susceptible to the chosen antibiotic.[22] This method is known for being inexpensive and simple, but it requires a minimum of 16–24 hours to grow the bacteria on the plate.[26, 27] This method has been adapted for automation, but it still requires at least 6 hours for bacterial growth.[28] The commercially available Etest works similarly to the disk diffusion method. The Etest uses plastic strips that contains antibiotic in a gradient along the strip that are placed on agar plates and incubated to promote bacterial growth. The Etest is more quantitative than the disk diffusion method and offers improved inter-laboratory precision.[22, 29] However, the growth of bacteria still requires about 24 hours to produce results.[30] Collectively, current susceptibility tests provide important information regarding the concentration and type of antibiotic that can be used for a given bacteria, but they suffer from long incubation times, low precision among the different types of tests, and qualitative readout. [23, 31, 32]

To overcome the shortcomings of current susceptibility tests, we report here the development of a rapid test based on ATP, which has been previously used to detect the presence of living bacteria for cleanliness purposes in both the food and medical industries.[33–35] Intracellular ATP, typically at concentrations of 1–5 mM,[36–38] has also been used in susceptibility testing, but requires lysing the bacterial cells to access the intracellular stores.[39] Importantly, extracellular ATP has been detected at concentrations ranging from 15 nM to 1.9 mM depending on the releasing bacterial strain and environment.[36, 40, 41] Extracellular ATP is often viewed as a signaling molecule for communication between cells.[42, 43] It was recently reported that bacteria release ATP into the extracellular matrix during the log phase of bacterial growth. The extracellular ATP then decreases due to degradation or hydrolization by the bacteria after they reach the stationary phase of growth. This trend of increasing, then decreasing, extracellular ATP is only seen when the bacteria are alive and growing.[36] We use these features of bacterial growth (in the form of a measurement of sample optical density, OD600) and ATP levels to determine the susceptibility of bacteria to an antibiotic within minutes of adding the antibiotic to a growing bacterial culture, thus providing a quantitative susceptibility test that is also rapid.

## Materials and methods

### Growth media & agar plate preparation

Lysogeny broth (LB, EMD Chemicals, Darmstadt, Germany) was prepared by dissolving 3.0 g of LB Broth–Lennox pellets in 300 mL of distilled and deionized water (DDW). The LB solution was then autoclaved for 45 minutes at 121°C and allowed to cool prior to addition of antibiotics. Agar plates were created using the same procedure as the LB, but 2.25 g of agar (laboratory grade, Fisher Scientific, Fair Lawn, NJ) were added prior to autoclaving.

### Antibiotic reagents

Kanamycin sulfate (USP grade) and 10 mg/mL Gentamicin reagent solution were purchased from Gibco by Life Technologies (Grand Island, NY). Levofloxacin (HPLC, ≥ 98.0%), chloramphenicol (water soluble) and tetracycline hydrochloride, were purchased from Sigma Life Science (St. Louis, MO). After cooling of the LB broth, 50 mg of kanamycin were added to each liter of LB solution, resulting in a final concentration of 100 μM for kanamycin resistant (KanR) *E*. *coli*. For chloramphenicol, 5 mg were added per liter of LB solution, resulting in a concentration of 15 μM for (chloramphenicol resistant (CmpR) *B*. *subtilis* (selective LB) studies. The use of the drug to which the bacteria are resistant was to ensure that only the bacteria of interest were growing.

### Bacterial strains

KanR *E*. *coli* was obtained from Dr. David P. Weliky[44] and CmpR *B*. *subtilis* was obtained from Dr. Lee R. Kroos,[45] both from Michigan State University. A levofloxacin–resistant strain of *E*. *coli* was created by growing KanR *E*. *coli* in 10 mL of selective LB containing 50 mg/L kanamycin for 6 hours and plating 1 mL of the culture onto a selective agar plate. Selective LB and selective agar are composed of LB media containing an antibiotic so only the bacteria of interest are permitted to grow. A 22 mm disk cut from P5 filter paper (Fisherbrand, Pittsburgh, PA) was placed in a solution of 10 mg/L levofloxacin. This filter paper disk was then placed at the center of the selective agar plate and the plate was incubated upside down at 37°C overnight to promote growth. The filter paper disk was used to create a gradient of levofloxacin that decreased in concentration as the distance from the disk increased. After overnight growth, bacteria that grew closest to the filter paper disk were transferred by an inoculating loop to 10 mL of new selective LB. This process was repeated until the bacteria could grow successfully in a solution containing 5 mg/L of levofloxacin. The bacteria were made into a glycerol stock and stored at -80°C. To prepare a glycerol stock solution, a bacterial colony from an agar plate was transferred to 10 mL of selective LB. The bacteria were grown to mid-logarithmic phase (5 hours). Glyercol (spectrophotometric grade, 99.5+%) was purchased from Aldrich (Milwaukee, WI) and prepared as an 80% (v/v) glycerol solution in DDW and autoclaved at 121°C for 45 minutes. Bacterial stock solutions were prepared in a sterile tube by using 800 μL of the mid-logarithmic phase bacteria and 200 μL of the 80% glycerol solution. The stock solutions were briefly mixed by vortexing and then stored at -80°C.

### Sample preparation

**Fig 1:**
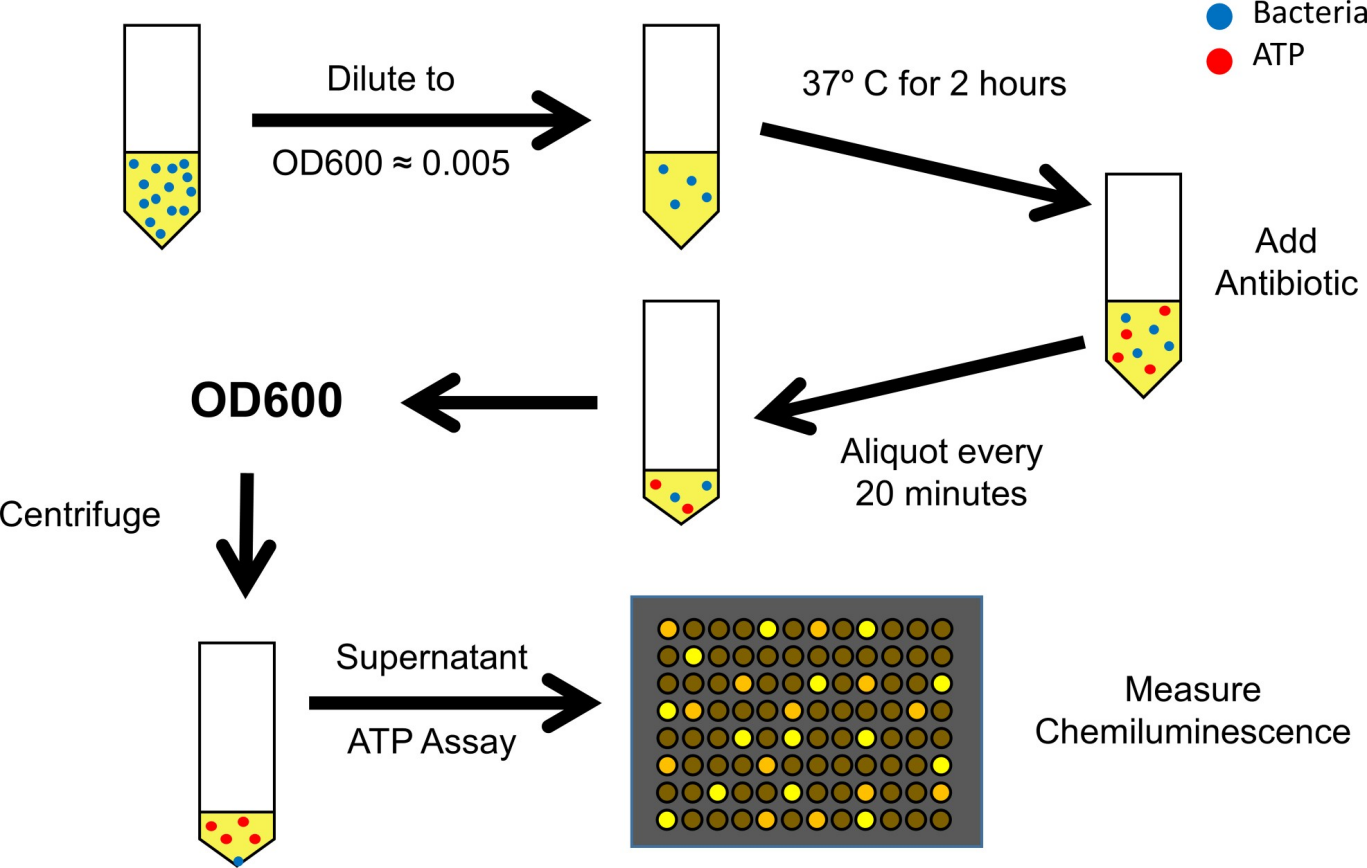
The overall procedure for the susceptibility assay. The overall assay procedure involves the growth of a single colony of bacteria overnight in selective media at 37°C with shaking (not shown). The OD600 is measured and a diluted culture of 10 mL is prepared by dilution to an OD600 of approximately 0.005. After 2 hours of growth at 37°C, 1 mL of the diluted culture is removed and the remainder of the diluted culture is mixed with the antibiotic to which the bacteria are susceptible. Aliquots are removed at 20, 40, and 60 minutes after adding the antibiotic for OD600 determination; next, each aliquot is centrifuged at 30,000*g* for 30 seconds. The supernatant is transferred to a black bottom 96 well plate for the quantitative determination of ATP.

This procedure (Fig 1) was adapted from Mempin et al. [36] A sterile inoculating loop was touched against the bacteria-glycerol stock and plated on an agar plate containing the drug to which the bacteria are resistant. This plate was inverted and incubated overnight for *E*. *coli* or 48 hours for *B*. *subtilis*. One colony was scratched from the plate using a sterile pipette tip and the pipette tip was placed in a culture tube containing 2 mL of selective LB containing 50 mg/L kanamycin or 5 mg/L chloramphenical to ensure that only KanR *E*.*coli* or CmpR *B*. *subtilis* could grow, respectively. The culture tube was incubated at 200 rpm and 37°C overnight. Bacterial culture incubation was performed using a Talboys Professional Incubating Orbital Minishaker (Talboys, Thorofare, NJ). The OD600 of the overnight culture was measured and a diluted culture was prepared to an OD600 ~ 0.005. The diluted culture was grown for 2 hours at 200 rpm and 37°C. After 2 hours, 1 mL of culture was aliquoted and the drug of interest was added to pharmacological concentration (5 mg/L for levofloxacin, and chloramphenicol; 6–10 mg/L for gentamicin; or 50 mg/L for kanamycin). Once the drug of interest was added, the culture resumed incubation at 200 rpm and 37°C. Additional 1 mL samples were aliquoted at 20, 40, and 60 minutes after introduction of the drug of interest. Aliquots were stored at 4°C until the operator was ready to measure the OD600 of the bacterial culture and the extracellular concentration of ATP in the supernatant.

### Determination of OD600 and ATP

The relative amount of bacteria in solution was measured by OD600. OD600 is an absorbance measurement at a wavelength of 600 nm. A sample of 150 μL was pipetted from the aliquots and transferred to a clear bottom 96 well plate. The remaining volume of the aliquots was centrifuged at 30,000*g* for 30 seconds. The supernatant was transferred to a new tube and stored at 4°C until ATP measurements were performed. The ATP concentrations of each aliquot were measured using the luciferin-luciferase assay. This assay is a chemiluminescence measurement where the amount of light produced is dependent on the concentration of ATP in the sample. The luciferin-luciferase solution was made by dissolving 2 mg of potassium luciferin (Gold Biotechnology, St. Louis, MO) and 10 mg of firefly lantern extract (Sigma) into 5 mL of DDW. Extra luciferin-luciferase reagent was stored at -20°C. In a black bottom 96 well plate, 150 μL of sample were added to a well and 20 μL of the luciferin-luciferase solution were added and mixed directly before measuring luminescence. The concentration of ATP (Adenosine 5’-triphosphatte disodium salt hydrate (Grade I, ≥ 99%), Sigma) was determined by comparing the luminescence levels with an ATP standard curve. An ATP working solution was prepared at a concentration of 1,000 nM using standard ATP in DDW. Serial dilution was performed using selective LB to create concentrations for the standard curve. The extracellular ATP concentration is dependent on the number of bacteria cells in culture, therefore the ATP/OD600 ratio was used as an indicator of cell status. The ATP/OD600 ratio can vary due to the age of bacteria, LB, or agar plates. To account for these variables, the ATP/OD600 ratios were normalized against the ATP/OD600 ratio of the control before the drug of interest was added. The ATP/OD600 ratios at a given time point were compared with the control at the same time point to determine if there was a significant difference (α = 0.05) between the ratios. All ATP and OD600 measurements were performed using a Flexstation 3 (Molecular Devices, Sunnyvale, CA). Centrifugation was performed using a Sorvall ST 8R Centrifuge (Thermo Scientific, Waltham, MA).

## Results and discussion

### ATP/OD600 of Gram-negative *E*. *coli*

Bacterial growth occurs in three phases; (1) an initial lag phase, where the bacteria adjust to environmental factors and are not dividing at a significant rate, (2) a log phase (often called the logarithmic or exponential phase) where the bacterial cells double at a constant, exponential rate, and (3) a stationary phase where population growth is steady. It was recently reported that bacteria release ATP into the extracellular environment during growth until the late logarithmic/early stationary phase. During the stationary phase, the ATP concentration decreased, possibly due to hydrolysis of the ATP on the surface of living bacteria (when bacteria were killed, the ATP remained constant as opposed to decreasing).[36] Of course, during this period of rapid cell growth, there is also an increase in the OD600 of the bacterial solution. A result of the ATP release and OD600 values is an initial increase in the ATP/OD600 ratio, followed by an eventual decrease as the bacteria approach the stationary phase (due to the ATP depletion and a constant OD600). Importantly, this pattern of increasing and decreasing ATP/OD600 values was measured for many different types of gram-positive and gram-negative bacteria. Based on these results, we anticipated that the addition of an antibiotic to the bacterial solution at the ATP/OD600 maximum value could be used as a new susceptibility test. Specifically, at the peak ratio, we hypothesized that the bacteria were now in the stationary phase (i.e., alive, but not growing) and ATP values would begin decreasing. The addition of an effective antibiotic would lyse the bacteria, resulting in an increase in release of the intracellular ATP found in the bacteria to the extracellular matrix. Because the OD600 is independent of whether a bacterial cell is living or dead, the result of an effective antibiotic would be an increase in the ATP/OD600 ratio over time. To ensure maximum efficiency of this method, however, it would be necessary to reach the maximum ATP/OD600 value; adding the antibiotic prior to this point would make it difficult to differentiate between ATP release due to cell growth and ATP release due to cell lysis.

**Fig 2:**
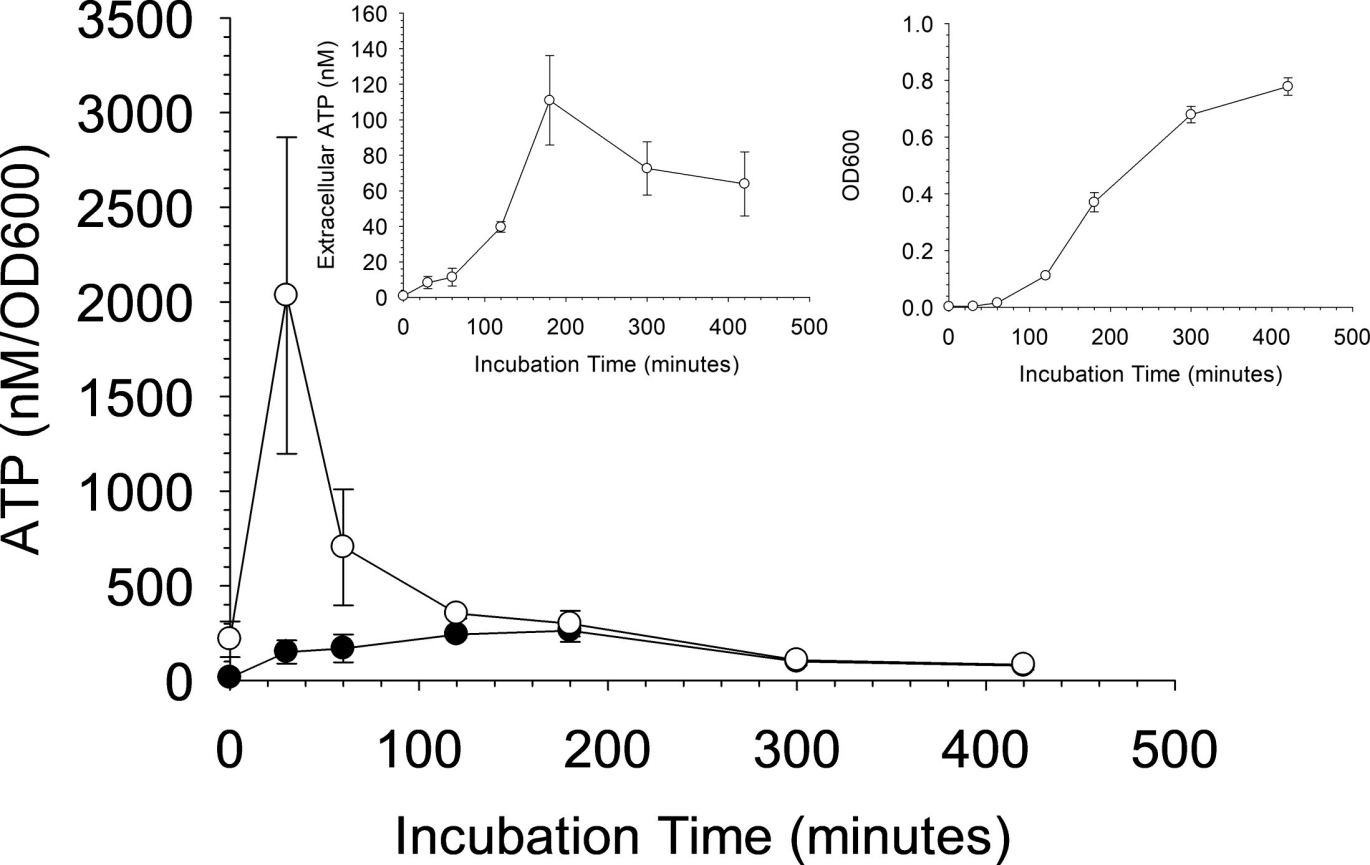
Evaluation of the extracellular ATP and optical density during growth of *E*. *coli*. ATP/OD600 curve for gram-negative *E*. *coli* with (open circles) and without (dark circles) the background absorbance caused by the 96 well plate and growth media. Inlayed graphs show the individual extracellular ATP (left inlay) and OD600 (right inlay) curves. The OD600 curve does not contain the background absorbance. n = 3; error = standard deviation. Note the shift in the peak ATP:OD600 maximum value for the measurements that include the background subtraction.

Fig 2 shows the ATP release and OD600 from kanamycin-resistant *Escherichia coli (E*. *coli)*. The ATP/OD600 peak ratio is estimated to occur around 180 minutes (filled circles), similar to previous reports. However, in these experiments, we also subtracted background absorbance of the 96 well plate and the growth media and discovered there is a period where small amounts of bacteria seem to result in a large increase in extracellular ATP concentration. For this strain of *E*. *coli*, this period of high ATP releases occurs around 30 minutes (open circles). This increase would normally be masked by the background absorbance of the plate and media because the increased absorbance from bacteria is small compared to the background absorbance of the well plate and the media. Thus, background subtraction shifted the peak ATP/OD600 ratio from ~ 180 minutes to ~ 30 minutes. This finding is important because, as described above, effects from added antibiotics should be detectable if the ATP/OD600 values have reached a maximum value.

### Antibiotic effect on *E*. *coli* ATP/OD600

**Fig 3:**
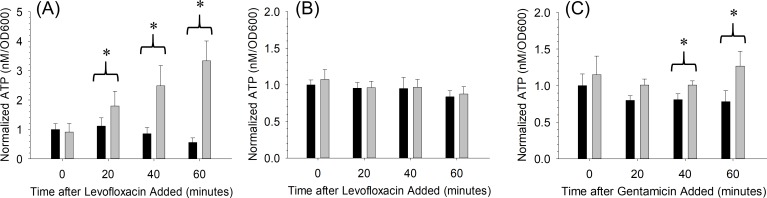
Normalized ATP/OD600 levels showing susceptibility and resistance of *E*. *coli* to bactericidal antibiotics. *E*. *coli* bacteria were preincubated for 2 hours with the antibiotic kanamycin (50 mg/L, black bars), which permits selective growth, before adding an antibiotic to which the bacteria are susceptible (gray bars). (A) ATP/OD600 levels of kanamycin-resistant *E*. *coli* with kanamycin (50 mg/L) versus kanamycin (50 mg/L) and the antibiotic to which the bacteria are susceptible, levofloxacin (5 mg/L). (B) ATP/OD600 levels of kanamycin/levofloxacin-resistant *E*. *coli* with kanamycin (50 mg/L) versus kanamycin (50 mg/L) and levofloxacin (5 mg/L). (C) ATP/OD600 levels of kanamycin/levofloxacin-resistant *E*. *coli* with kanamycin (50 mg/L) versus kanamycin (50 mg/L) and the antibiotic to which the bacteria are susceptible, gentamicin (6 mg/L). n = 3; error = standard deviation; * p < 0.05.

To measure the effects of an antibiotic on a bacteria-containing sample, the fluoroquinolone bactericidal antibiotic, levofloxacin, was added at a pharmacological concentration of 13.8 μM (5 mg/L) to the kanamycin-resistant *E*. *coli* culture after 2 hours of uninhibited growth. As mentioned above, the antibiotic to which the bacteria are susceptible, levofloxacin in this example, could have been added after 30 minutes, but to establish antibiotic efficacy, we chose to use 2 hours for these first set of experiments to ensure that the ATP/OD600 ratio value was low to ensure optimal sensitivity in a subsequent increase due to bacterial lysis. A significant difference in the ATP/OD600 ratio (α = 0.05) between the bacteria samples exposed to kanamycin and levofloxacin (1.80 +/- 0.50) and kanamycin alone (1.12 +/- 0.28) was measurable 20 minutes after adding the antibiotic (Fig 3A). This difference in the increase in ATP/OD600 between the samples is believed to be associated with the susceptibility of bacteria to that antibiotic. The addition of levofloxacin to the kanamycin-resistant strain resulted in a lysis of the bacteria cells, thus releasing intracellular stores of ATP while the OD600 remains constant.

To ensure that the measureable difference in ATP/OD600 between the levofloxacin-containing samples and control samples was due to antibiotic susceptibility and not just the presence of the added antibiotic, the kanamycin-resistant *E*. *coli* was made levofloxacin-resistant. The assay was then repeated using the *E*. *coli* that was resistant to both kanamycin and levofloxacin-resistant. As shown in Fig 3B, no significant difference was measured over the same period as that shown in Fig 3A. Finally, the assay was repeated using the kanamycin and levofloxacin-resistant *E*.*coli*, but this time the strain was challenged with the aminoglycoside bactericidal antibiotic, gentamicin at pharmacological concentrations 12.6 μM (6 mg/L). A significant difference is measured between the gentamicin and control sample within 40 minutes of challenging the bacteria (Fig 3C), thus providing evidence that only an antibiotic to which the bacteria are susceptible will affect the ATP/OD600 of the bacterial samples. The longer time required to measure the significant difference using the gentamicin is possibly caused by the differences in the mechanisms of action of the antibiotics themselves. The above results show the ability of this method to determine antibiotic susceptibility of bactericidal antibiotics on KanR *E*. *coli*. This method could also work for bacteriostatic antibiotics; however, it may take longer after the addition of the antibiotic before a statistically significant difference in ATP/OD600 ratios can be measured when compared to the controls. The ATP/OD600 ratio, of the bacteria challenged with the bacteriostatic antibiotic, would not be increasing as rapidly since the bacteriostatic antibiotic would not be causing lysis at a high rate like a bactericidal antibiotic. The difference in ratios would not be detectable until the ATP/OD600 ratio of control bacteria cultures deplete the extracellular ATP in the late logarithmic/early stationary phase of growth.

**Fig 4:**
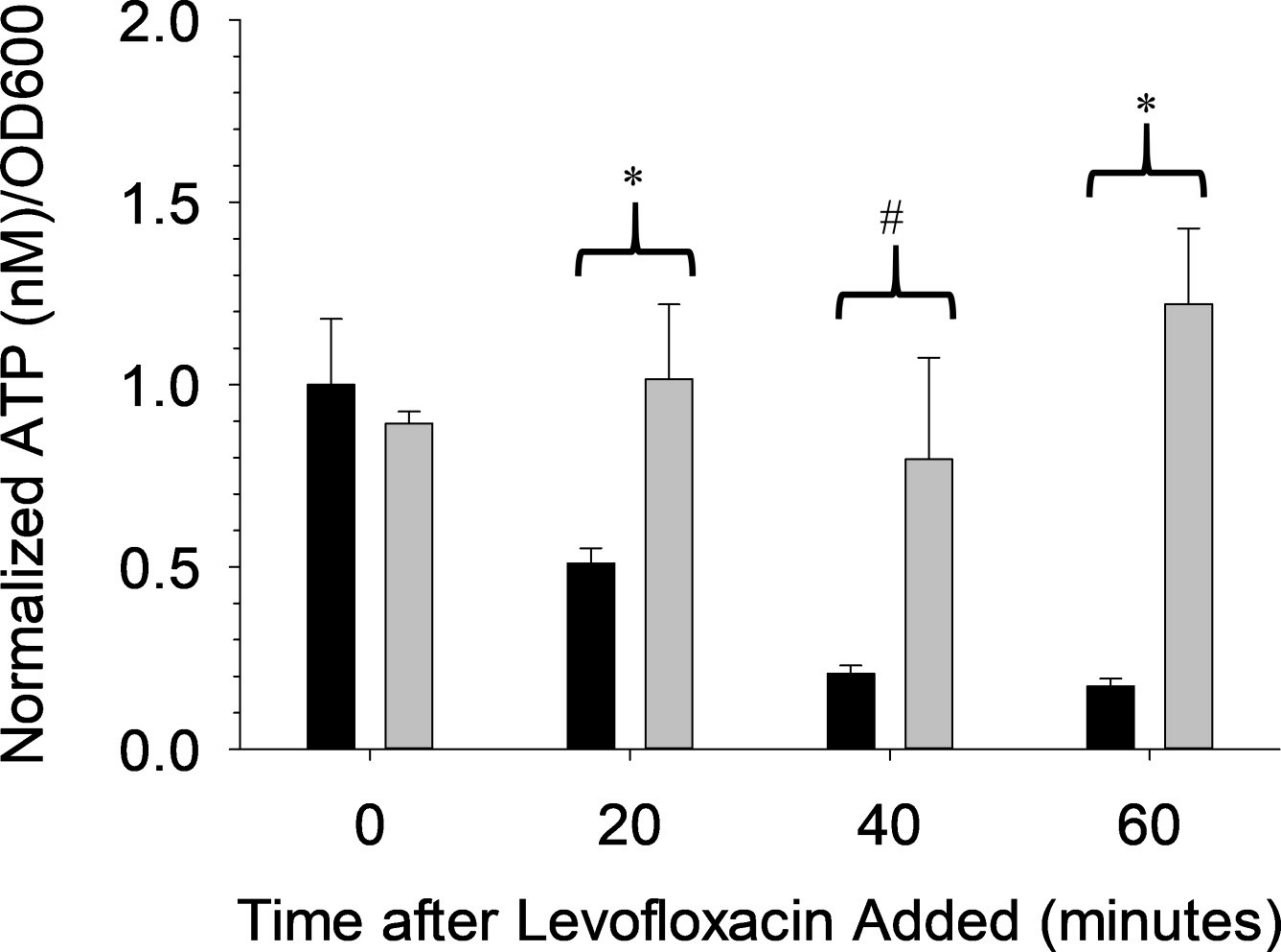
Normalized ATP/OD600 levels showing susceptibility of *E*. *coli* to a bactericidal antibiotic with only a 30 minute pre-incubation. The *E*. *coli* bacteria were preincubated for only 30 minutes with the antibiotic kanamycin (50 mg/L, black bars) which permits selective growth, before adding the antibiotic to which the bacteria are susceptible, levofloxacin (gray bars). Levofloxacin (5 mg/L) along with the antibiotic that permits selective growth, were compared against a control containing only the antibiotic that permits selective growth. n = 3; error = standard deviation; * p < 0.05; # p < 0.10.

The data in Fig 3 provide evidence that antibiotic efficacy can be measured with high significance levels. However, one of the objectives of our studies was to provide a rapid antibiotic susceptibility test; therefore, the assay used to create Fig 3A was repeated with only a 30 minute growth period with kanamycin (as opposed to the 2 hour growth period prior to addition of the antibiotic to which the bacteria are susceptible used in Fig 3). Importantly, the data in Fig 4 show a significant difference (α = 0.05) in the ATP/OD600 ratio between levofloxacin-free solution (0.51 +/- 0.04) and levofloxacin-containing samples (1.02 +/- 0.20). By adding the antibiotic close to the maximum ATP/OD600 level on the growth curve in Fig 2 (~ 30 minutes), the ATP/OD600 decreases rapidly; thus, an ineffective antibiotic would not have an effect on the sample and the ratio would continue to decrease. In contrast, the efficacy of the levofloxacin resulted in cell lysis, an increase in extracellular ATP and a subsequent higher ATP/OD600 value. Collectively, while the ATP/OD600 ratio absolute values are lower, the increase in the ratio is still indicative of an effective antibiotic and could lead to much earlier detection of antibiotic susceptibility.

### Gram-positive *B*. *Subtilis*

**Fig 5:**
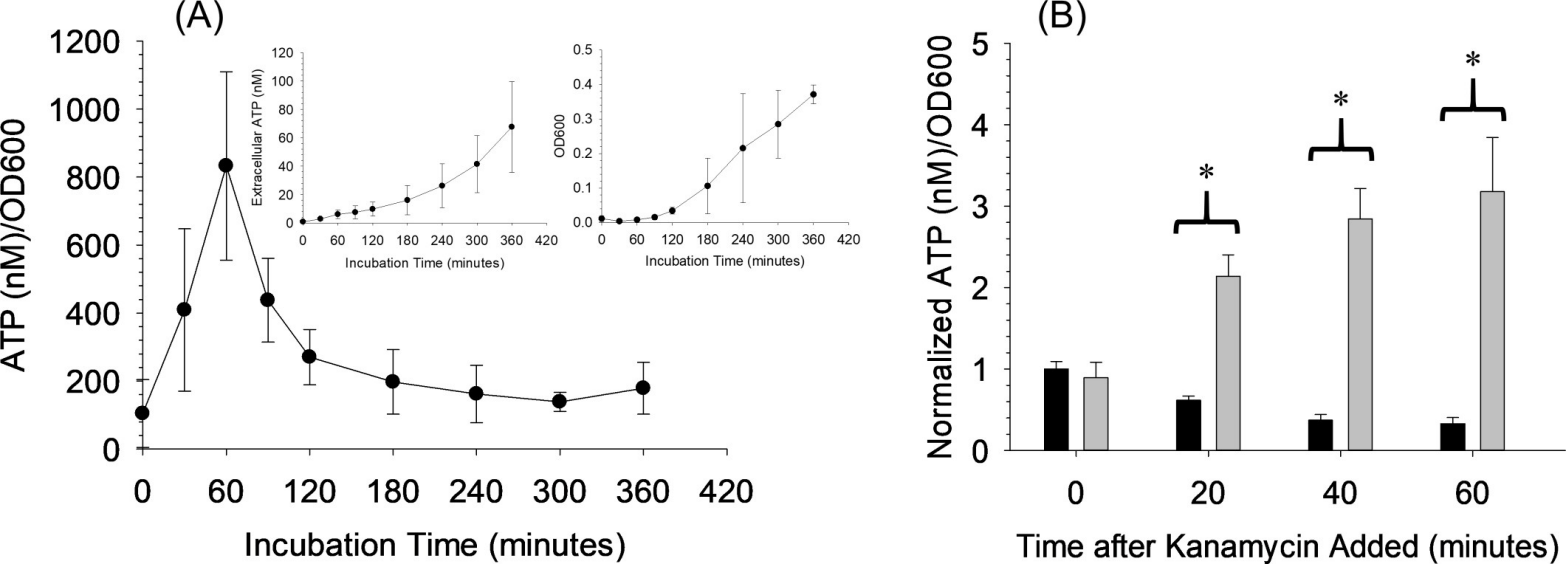
Results for gram-positive *B*. *subtilis*. (A) ATP/OD600 curve for gram-positive *B*. *subtilis*. Inlayed graphs show the individual extracellular ATP (left inlayed) and OD600 (right inlayed) curves. The OD600 curve does not contain the background absorbance. n ≤ 3; error = standard deviation. (B) Normalized ATP/OD600 graph showing susceptibility of *B*. *subtilis* to a bactericidal antibiotic. *B*. *subtilis* was preincubated for 2 hours with the antibiotic chloramphenicol (5 mg/L, black bars), which permits selective growth, before adding the antibiotic to which the bacteria are susceptible, kanamycin (gray bars). Kanamycin (50 mg/L), along with the antibiotic that permits selective growth, was compared against a control containing only the antibiotic that permits selective growth. n = 3; error = standard deviation; * p < 0.05.

The kanamycin-resistant strain of *E*. *coli* is a gram-negative strain. Therefore, to demonstrate that the reported method is also effective with gram-positive bacteria, we also tested *Bacillus subtilis (B*. *subtilis)*. Following a similar protocol for the gram-negative *E*. *coli* strain, the ATP/OD600 growth curve was measured to determine the required pre-incubation time to pass the peak of maximum ATP/OD600 for Chloramphenicol-resistant *B*. *subtilis*. The maximum ATP/OD600 for *B*. *subtilis* occurred 60 minutes after starting the incubation (Fig 5A). The inlayed graphs show that this peak is the result of an increase in ATP by a comparatively small number of bacteria, the same trend observed for *E*. *coli*. *E*. *coli* Chloramphenicol was allowed to incubate with the Chloramphenicol-resistant *B*. *subtilis* for 120 minutes to remain consistent with the assay for *E*. *coli*. kanamycin (50 mg/L or 103.2 μM) was used as the antibiotic to which the bacteria are susceptible in this study and, as shown in Fig 5B, an increase in the ATP/OD600 ratio was measured for the bacteria exposed to kanamycin (2.14 +/- 0.26) in comparison to those bacteria subjected to Chloramphenicol alone (0.62 +/- 0.05). Importantly, this difference is measured at 20 minutes after adding the kanamycin, thus demonstrating potential as a rapid susceptibility assay regardless of gram-negative or gram-positive classification. Bacterial strains with slower growth like CmpR *B*. *subtilis*, when compared to KanR, can still be evaluated for antibiotic susceptibility, but the method requires a longer preincubation time before the maximum ATP/OD600 is reached and more time may be required before a statistical difference from controls can be measured.

### Mixed bacterial culture

**Fig 6:**
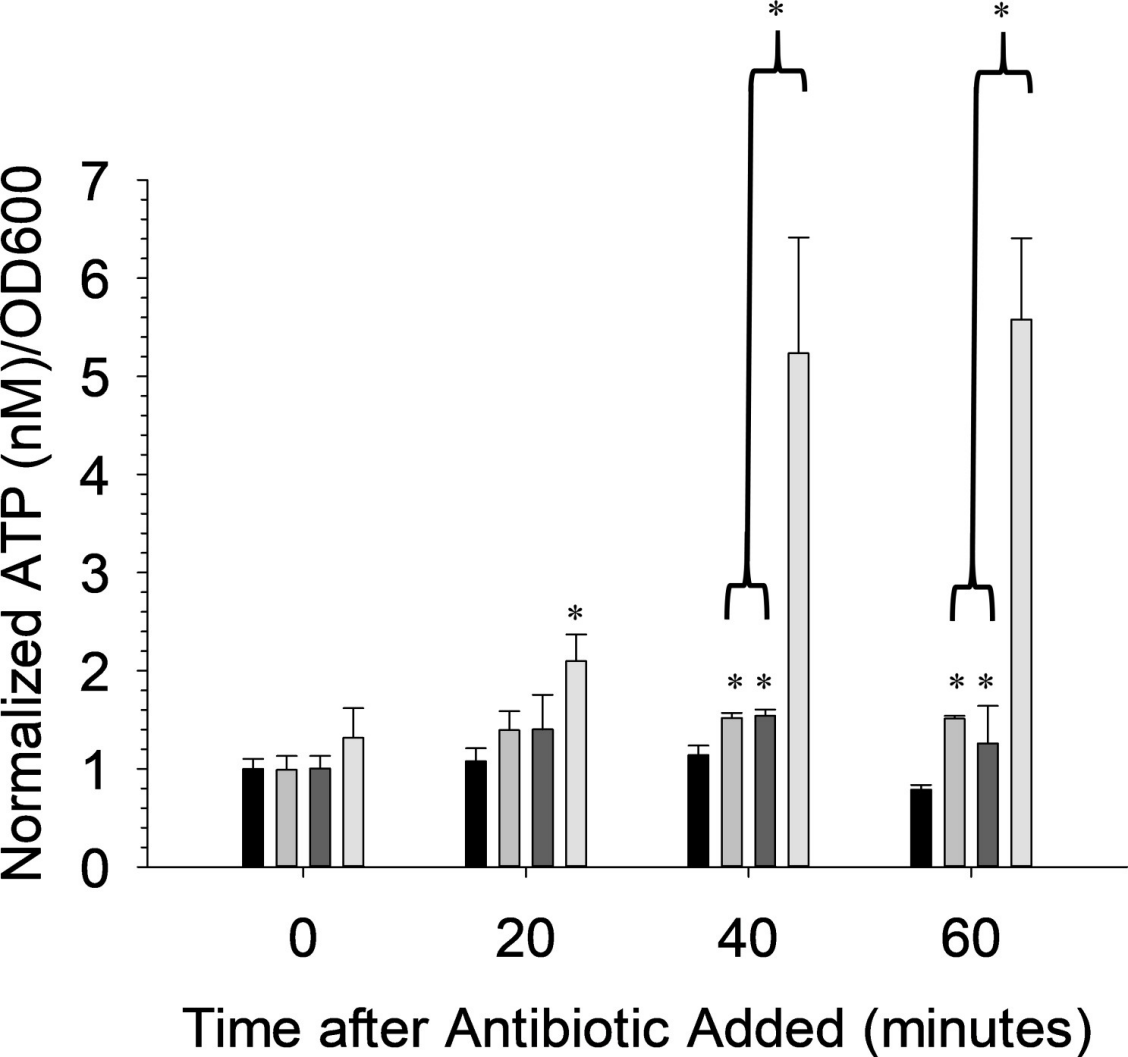
Normalized ATP/OD600 levels showing susceptibility of kanamycin-resistant *E*. *coli* and chloramphenicol-resistant *B*. *subitilis* mixed culture to bactericidal antibiotics. The bacterial mixed culture was preincubated for 2 hours before adding the antibiotic. Chloramphenicol (5 mg/L) (light gray), kanamycin (50 mg/L) (dark gray), or gentamicin (10 mg/L) (white) were compared against a control containing no antibiotic (black). n = 3; error = standard deviation; * p < 0.05.

A potential high-impact use for this assay is to test an antibiotic against an infection where the identity of the bacteria causing the infection is unknown. Multiple bacteria strains could be present at the infection site, even if only one strain is causing the infection. To test such a system, both the kanamycin-resistant *E*. *coli* and the Chloramphenicol-resistant *B*. *subtilis* were mixed in culture equally to an OD600 ~ 0.005. Both strains were incubated in media without an antibiotic that permits selective growth to ensure growth that would be uninhibited by antibiotics. After a pre-incubation time of 2 hours, either Chloramphenicol (5 mg/L or 15.5 μM), kanamycin (50 mg/L or 103.2 μM), or Gentamicin (10 mg/L or 20.9 μM) was added to the mixed culture. While both strains of bacteria are susceptible to Gentamicin, each is only susceptible to one of the other antibiotics. The data in Fig 6 show a significant difference in ATP/OD600 ratio when Gentamicin is used as the antibiotic to which the bacteria are susceptible in comparison to controls; also, this difference could be quantitatively measured in 20 minutes. There were some slight effects from the other two antibiotics, but these changes were not as significant and required 40 minutes to be measured. Importantly, the data in Fig 6 demonstrate that this method could be used to determine antibiotic efficacy against an unknown bacterium within 20–40 minutes.

## Conclusions

Increasing cost of producing a drug, and the speed at which bacteria become resistant to new drug candidate molecules, is resulting in a dangerous situation where certain bacterial strains are becoming resistant to all available antibiotics. There is a need for rapid diagnostic tools to facilitate drug discovery and clinical assays. Here, we have described a method to determine antibiotic efficacy against both gram-negative and gram-positive bacteria. Unlike other susceptibility tests, our test is quantitative and results can be obtained in as little as 20 minutes after the addition of an antibiotic to a growing culture. The method is simple, requiring only an optical density measurement at 600 nm (OD600) and a determination of ATP requiring the well-established luciferase assay. Both of these components can be easily measured with a standard multi-modal plate reader, which can also be used for the established 96-well plate microdilution antibiotic susceptibility test. The test could easily be multiplexed to determine efficacy of multiple antibiotics against multiple strains simultaneously.

## References

[1] DiMasi JA, Grabowski HG, Hansen RW. Innovation in the pharmaceutical industry: New estimates of R&D costs. Journal of health economics. 2016;47:20–33. Epub 2016/03/02. 10.1016/j.jhealeco.2016.01.012 . 26928437

[2] Hay M, Thomas DW, Craighead JL, Economides C, Rosenthal J. Clinical development success rates for investigational drugs. Nature biotechnology. 2014;32(1):40 10.1038/nbt.2786 24406927

[3] Getz KA, Wenger J, Campo RA, Seguine ES, Kaitin KI. Assessing the impact of protocol design changes on clinical trial performance. American journal of therapeutics. 2008;15(5):450–7. 10.1097/MJT.0b013e31816b9027 18806521

[4] DiMasi JA, Feldman L, Seckler A, Wilson A. Trends in risks associated with new drug development: success rates for investigational drugs. Clinical pharmacology and therapeutics. 2010;87(3):272–7. Epub 2010/02/05. 10.1038/clpt.2009.295 . 20130567

[5] Berndt ER, Nass D, Kleinrock M, Aitken M. Decline in economic returns from new drugs raises questions about sustaining innovations. Health affairs (Project Hope). 2015;34(2):245–52. Epub 2015/02/04. 10.1377/hlthaff.2014.1029 . 25646104

[6] Scannell JW, Blanckley A, Boldon H, Warrington B. Diagnosing the decline in pharmaceutical R&D efficiency. Nature reviews Drug discovery. 2012;11(3):191 10.1038/nrd3681 22378269

[7] Waring MJ, Arrowsmith J, Leach AR, Leeson PD, Mandrell S, Owen RM, et al An analysis of the attrition of drug candidates from four major pharmaceutical companies. Nature reviews Drug discovery. 2015;14(7):475 10.1038/nrd4609 26091267

[8] Munos B. Lessons from 60 years of pharmaceutical innovation. Nature reviews Drug discovery. 2009;8(12):959–68. Epub 2009/12/02. 10.1038/nrd2961 . 19949401

[9] Taubes G. The bacteria fight back. Science (New York, NY). 2008;321(5887):356–61. Epub 2008/07/19. 10.1126/science.321.5887.356 . 18635788

[10] Blair JMA, Webber MA, Baylay AJ, Ogbolu DO, Piddock LJV. Molecular mechanisms of antibiotic resistance. Nature Reviews Microbiology. 2014;13:42 10.1038/nrmicro3380 25435309

[11] van Duijkeren E, Schink AK, Roberts MC, Wang Y, Schwarz S. Mechanisms of Bacterial Resistance to Antimicrobial Agents. Microbiology spectrum. 2018;6(1). Epub 2018/01/13. 10.1128/microbiolspec.ARBA-0019-2017 . 29327680 PMC11633570

[12] Botts RT, Apffel BA, Walters CJ, Davidson KE, Echols RS, Geiger MR, et al Characterization of Four Multidrug Resistance Plasmids Captured from the Sediments of an Urban Coastal Wetland. Frontiers in Microbiology. 2017;8(1922). 10.3389/fmicb.2017.01922 29067005 PMC5641379

[13] Miller JH, Novak JT, Knocke WR, Pruden A. Survival of Antibiotic Resistant Bacteria and Horizontal Gene Transfer Control Antibiotic Resistance Gene Content in Anaerobic Digesters. Frontiers in Microbiology. 2016;7(263). 10.3389/fmicb.2016.00263 27014196 PMC4781833

[14] Juhas M. Horizontal gene transfer in human pathogens. Critical Reviews in Microbiology. 2015;41(1):101–8. 10.3109/1040841X.2013.804031 23862575

[15] Jutkina J, Rutgersson C, Flach C-F, Joakim Larsson DG. An assay for determining minimal concentrations of antibiotics that drive horizontal transfer of resistance. Science of The Total Environment. 2016;548–549:131–8. 10.1016/j.scitotenv.2016.01.044 26802341

[16] Meir-Gruber L, Manor Y, Gefen-Halevi S, Hindiyeh MY, Mileguir F, Azar R, et al Population screening using sewage reveals pan-resistant bacteria in hospital and community samples. PloS one. 2016;11(10):e0164873 10.1371/journal.pone.0164873 27780222 PMC5079554

[17] Ghafur A, Lakshmi V, Kannain P, Thirunarayan M. Emergence of Pan drug resistance amongst gram negative bacteria! The First case series from India. Journal of Microbiology and Infectious Diseases. 2014;4(03).

[18] Zowawi HM, Forde BM, Alfaresi M, Alzarouni A, Farahat Y, Chong T-M, et al Stepwise evolution of pandrug-resistance in Klebsiella pneumoniae. Scientific reports. 2015;5:15082 10.1038/srep15082 26478520 PMC4609946

[19] Jooma S. Executive action to combat the rise of drug-resistant bacteria: is agricultural antibiotic use sufficiently addressed? Journal of Law and the Biosciences. 2015;2(1):129–38. 10.1093/jlb/lsv005 27774190 PMC5033552

[20] Obama B. Executive Order—combating antibiotic-resistant bacteria. The White House: Office of the Press Secretary 2014.

[21] House W. National action plan for combating antibiotic-resistant bacteria. Washington, DC 2015.

[22] Jorgensen JH, Ferraro MJ. Antimicrobial susceptibility testing: a review of general principles and contemporary practices. Clinical infectious diseases: an official publication of the Infectious Diseases Society of America. 2009;49(11):1749–55. Epub 2009/10/28. 10.1086/647952 . 19857164

[23] Gupta P, Khare V, Kumar D, Ahmad A, Banerjee G, Singh M. Comparative Evaluation of Disc Diffusion and E-test with Broth Micro-dilution in Susceptibility testing of Amphotericin B, Voriconazole and Caspofungin against Clinical Aspergillus isolates. Journal of Clinical and Diagnostic Research: JCDR. 2015;9(1):DC04–DC7. 10.7860/JCDR/2015/10467.5395 PubMed PMID: PMC4347075. 25737984 PMC4347075

[24] Prüller S, Frömke C, Kaspar H, Klein G, Kreienbrock L, Kehrenberg C. Recommendation for a standardised method of broth microdilution susceptibility testing for porcine Bordetella bronchiseptica. PloS one. 2015;10(4):e0123883 10.1371/journal.pone.0123883 25910232 PMC4409320

[25] Leong C, Buttafuoco A, Glatz M, Bosshard PP. Antifungal susceptibility testing of Malassezia spp. with an optimized colorimetric broth microdilution method. Journal of clinical microbiology. 2017;55(6):1883–93. 10.1128/JCM.00338-17 28381607 PMC5442545

[26] Buzón-Durán L, Capita R, Alonso-Calleja C. Antibiotic susceptibility of methicillin-resistant staphylococci (MRS) of food origin: A comparison of agar disc diffusion method and a commercially available miniaturized test. Food Microbiology. 2018;72:220–4. 10.1016/j.fm.2017.11.018 29407401

[27] Matuschek E, Brown DFJ, Kahlmeter G. Development of the EUCAST disk diffusion antimicrobial susceptibility testing method and its implementation in routine microbiology laboratories. Clinical Microbiology and Infection. 2014;20(4):O255–O66. 10.1111/1469-0691.12373 24131428

[28] Hombach M, Jetter M, Blöchliger N, Kolesnik-Goldmann N, Böttger EC. Fully automated disc diffusion for rapid antibiotic susceptibility test results: a proof-of-principle study. Journal of Antimicrobial Chemotherapy. 2017;72(6):1659–68. 10.1093/jac/dkx026 28333189

[29] Ranque S, Lachaud L, Gari-Toussaint M, Michel-Nguyen A, Mallié M, Gaudart J, et al Inter-laboratory reproducibility of Etest amphotericin-B and caspofungin yeast susceptibility testing and comparison with the CLSI method. Journal of clinical microbiology. 2012:JCM. 00490–12. 10.1128/JCM.00490-12 PMC3405630 22553230

[30] Badiee P, Alborzi A. Susceptibility of clinical Candida species isolates to antifungal agents by E-test, Southern Iran: A five year study. Iranian journal of microbiology. 2011;3(4):183 22530086 PMC3330181

[31] Alberti MO, Hindler JA, Humphries RM. Performance of Etest for Antimicrobial Susceptibility Testing of Abiotrophia defectiva and Granulicatella Species. Journal of clinical microbiology. 2016;54(8):2194–6. 10.1128/JCM.00822-16 27280419 PMC4963500

[32] Ogata SK, Gales AC, Kawakami E. Antimicrobial susceptibility testing for Helicobacter pylori isolates from Brazilian children and adolescents: comparing agar dilution, E-test, and disk diffusion. Brazilian Journal of Microbiology. 2014;45:1439–48. 25763052 10.1590/s1517-83822014000400039 PMC4323321

[33] Bottari B, Santarelli M, Neviani E. Determination of microbial load for different beverages and foodstuff by assessment of intracellular ATP. Trends in Food Science & Technology. 2015;44(1):36–48. 10.1016/j.tifs.2015.02.012

[34] Amodio E, Dino C. Use of ATP bioluminescence for assessing the cleanliness of hospital surfaces: A review of the published literature (1990–2012). Journal of Infection and Public Health. 2014;7(2):92–8. 10.1016/j.jiph.2013.09.005 24231159

[35] Luo J, Liu X, Tian Q, Yue W, Zeng J, Chen G, et al Disposable bioluminescence-based biosensor for detection of bacterial count in food. Analytical Biochemistry. 2009;394(1):1–6. 10.1016/j.ab.2009.05.021 19464252

[36] Mempin R, Tran H, Chen C, Gong H, Kim Ho K, Lu S. Release of extracellular ATP by bacteria during growth. BMC Microbiology. 2013;13(1):301 10.1186/1471-2180-13-301 24364860 PMC3882102

[37] Schneider DA, Gourse RL. Relationship between growth rate and ATP concentration in Escherichia coli: a bioassay for available cellular ATP. The Journal of biological chemistry. 2004;279(9):8262–8. Epub 2003/12/13. 10.1074/jbc.M311996200 . 14670952

[38] Yaginuma H, Kawai S, Tabata KV, Tomiyama K, Kakizuka A, Komatsuzaki T, et al Diversity in ATP concentrations in a single bacterial cell population revealed by quantitative single-cell imaging. Scientific Reports. 2014;4:6522 10.1038/srep06522 https://www.nature.com/articles/srep06522#supplementary-information. 25283467 PMC4185378

[39] Ivančić V, Mastali M, Percy N, Gornbein J, Babbitt JT, Li Y, et al Rapid Antimicrobial Susceptibility Determination of Uropathogens in Clinical Urine Specimens by Use of ATP Bioluminescence. Journal of Clinical Microbiology. 2008;46(4):1213–9. 10.1128/JCM.02036-07 18272708 PMC2292911

[40] Ivanova EP, Alexeeva YV, Pham DK, Wright JP, Nicolau DV. ATP level variations in heterotrophic bacteria during attachment on hydrophilic and hydrophobic surfaces. International microbiology: the official journal of the Spanish Society for Microbiology. 2006;9(1):37–46. Epub 2006/04/26. . 16636988

[41] Iwase T, Shinji H, Tajima A, Sato F, Tamura T, Iwamoto T, et al Isolation and Identification of ATP-Secreting Bacteria from Mice and Humans. Journal of Clinical Microbiology. 2010;48(5):1949–51. 10.1128/JCM.01941-09 20305009 PMC2863903

[42] Trautmann A. Extracellular ATP in the Immune System: More Than Just a “Danger Signal”. Science Signaling. 2009;2(56):pe6-pe. 10.1126/scisignal.256pe6 19193605

[43] Hironaka I, Iwase T, Sugimoto S, Okuda K-i, Tajima A, Yanaga K, et al Glucose Triggers ATP Secretion from Bacteria in a Growth-Phase-Dependent Manner. Applied and Environmental Microbiology. 2013;79(7):2328–35. 10.1128/AEM.03871-12 23354720 PMC3623225

[44] Ratnayake PU, Ekanayaka EAP, Komanduru SS, Weliky DP. Full-Length Trimeric Influenza Virus Hemagglutinin II Membrane Fusion Protein and Shorter Constructs Lacking the Fusion Peptide or Transmembrane Domain: Hyperthermostability of the Full-Length Protein and the Soluble Ectodomain and Fusion Peptide Make Significant Contributions to Fusion of Membrane Vesicles. Protein expression and purification. 2016;117:6–16. 10.1016/j.pep.2015.08.021 PubMed PMID: PMC4684446. 26297995 PMC4684446

[45] Halder S, Parrell D, Whitten D, Feig M, Kroos L. Interaction of intramembrane metalloprotease SpoIVFB with substrate Pro-σK. Proceedings of the National Academy of Sciences. 2017;114(50):E10677–E86. 10.1073/pnas.1711467114 29180425 PMC5740621

